# Can a Moderate Physical Activity Program Delivered in Primary Care Improve the Ankle-Brachial Index in Patients with Asymptomatic Peripheral Arterial Disease?

**DOI:** 10.7759/cureus.91729

**Published:** 2025-09-06

**Authors:** Andreea Iana, Roxana Folescu, Daniela Gurgus

**Affiliations:** 1 Family Medicine Clinic, Center of Preventive Medicine, Victor Babeș University of Medicine and Pharmacy, Timisoara, ROU

**Keywords:** ankle-brachial index, future health, intervention effect, physical activity program, primary care

## Abstract

Objective

We aimed to assess whether there is a difference in the ankle-brachial index (ABI) in patients without known peripheral arterial disease (PAD) measured in the Romanian primary care at the General Practitioner (GP) office before and after six months of moderate physical activity. The second goal was to demonstrate the importance of implementing a moderate physical activity program that can be prescribed in primary care for patients above 50 years.

Methodology

The pilot study enrolled 96 patients without symptoms or known PAD, selected randomly from the general population, according to inclusion and exclusion criteria. We measured the ABI in the GP practice at enrolment in the study and six months after they underwent a GP-led moderate physical activity program. The study took place from August 2024 to March 2025.

Results

The mean ABI in the study group was 0.98 before physical activity and increased to 1.07 after the moderate physical activity program, with a significant analysis of variance (ANOVA) *P*-value < 0.001.

Patients with low ABI at baseline (<0.9) had improvements after the prescribed GP-led moderate physical activity program, which may indicate an intervention effect.

Conclusions

All participants showed improvements in ABI after the physical activity program. The physical activity program shows an additional effect on ABI, as assumed. Our study is a pilot intervention without a control group, providing preliminary data through a practical primary care approach.

Implementing a moderate physical activity program at the GP level could improve ABI in patients with asymptomatic PAD. Promoting patient education and training, particularly for those over 50, in the GP office can raise awareness about the benefits of regular physical activity and the importance of monitoring ABI.

## Introduction

Unhealthy lifestyles have a significant impact on morbidity and mortality, which could be prevented through health promotion, as data published by the Centers for Disease Control [1]. Health promotion enables people to increase their control over their health and its determinants [2].

It is important to make the population aware of physical activity in their daily routine, as it is essential in preventing coronary, cerebrovascular, and peripheral arterial disease (PAD). Only a few studies have tested GP-led physical activity programs in primary care offices in Romania.

It is important to recognize early the onset of lower extremity PAD because these patients are at higher risk of a cardiovascular or cerebrovascular event, as studied by Aboyans et al. [3], Criqui et al. [4], and Ness and Aronow [5]. Early recognition and adequate treatment significantly reduce associated risks and mortality, as documented by the European Society of Cardiology (ESC).

Its prevalence increases exponentially with age; by 55 years, and particularly in those 65 and older, prevalence can reach as high as 20%, as reported by Aboyans et al. in 2018 [3]. In 2011, the European Society of Cardiology (ESC) recognized that most patients with PAD are asymptomatic and remain so, making diagnosis difficult without specialized screening [6].

In elderly patients, PAD is a common manifestation of atherosclerosis, warranting particular attention to early diagnosis, as shown in previous studies [7,8]. Identifying resources in primary care and promoting GP-led physical activity programs are important components of prevention strategies.

Non-invasive diagnostic technique, such as ultrasound examination, was used to measure ABI, which is an accessible tool for the diagnosis of PAD and for monitoring patients with PAD [9]. PAD is often underdiagnosed, and a large number of individuals are not aware of the clinical symptoms associated with PAD [10]. Promoting GP-led physical activity programs in primary care and raising patient awareness of ABI improvements could help reduce the prevalence of PAD in the general population.

## Materials and methods

Study design and participants

This pilot research study took place in a General Practitioner (GP) and Internal Medical Clinic private office in Timisoara, Romania.

The study was conducted in compliance with good clinical practice, under approval number 12, with an inclusion protocol approved by the Ethics Committee on August 1, 2024. All participants provided their written consent before participation. The patients were selected according to inclusion and exclusion criteria from the general population who addressed the GP office. The study took place from August 2024 to March 2025.

The sample consisted of patients over 50 years of age without symptoms or a prior diagnosis of peripheral arterial disease. Exclusion criteria included cerebrovascular disease, diabetes mellitus, kidney or liver failure, malignancy, current smoking, daily alcohol consumption greater than 30 g, and an ABI below 0.8. An important exclusion criterion was the prior diagnosis of atherosclerotic cardiovascular disease. All patients in the study underwent ECG tests, and only the patients negative for coronary disease were included.

A total of 96 patients were enrolled in the study, including 49 men (51.04%) and 47 women (48.96%), with a mean age of 65 ± 4.52 years. Participants were randomly selected from the general population and met the eligibility criteria of age, absence of symptoms, and no prior diagnosis of peripheral arterial disease. 

Patients were assessed before and after the moderate physical activity program explained by the GP. All assessments were conducted in the GP’s office, and no control group was included.

Assessments

Participants’ demographic characteristics, including age, sex, weight, height, and body mass index (BMI), were recorded. Anthropometric measurements were taken with participants wearing light indoor clothing and no shoes. Body weight was measured to the nearest 0.1 kg (Salus, Milan, Italy), and height to the nearest 0.5 cm using a stadiometer (Salus). Waist circumference was measured with a measuring tape at the minimum circumference between the xiphoid process and the umbilicus. Venous blood samples were collected after an eight-hour fast.

ABI was measured in the GP office at study enrollment and again six months after completion of the moderate physical activity program. Measurements were performed using an Edan SD3 vascular Doppler (4 MHz, Milan, Italy). For accurate ABI assessment, patients were placed in the supine position. Both the leg and arm were positioned at heart level. Blood pressure was measured in both arms using a standard sphygmomanometer cuff appropriate for limb size, and the higher value was used for ABI calculation. The ABI was determined by dividing the higher systolic pressure at the ankle by that of the brachial artery. Measurements were repeated twice per site to ensure accuracy, and the highest pressures from the arm and ankle were used in the calculation. The normal ABI range was defined as 0.9-1.3

Interventions

All patients in the sample group participated in a six-month moderate aerobic training program, performed five times per week for 30 minutes per day, at a self-reported intensity of 60%-80% of their maximum heart rate.

It was explained that the training session should be preceded by 10-minute warm-up exercises for upper and lower limbs, at a maximal heart rate 20 beats/min higher than the heart rate at rest.

Recommended physical activities for the six-month training program included jogging, swimming, racket sports, brisk walking or treadmill walking on a slope, cycling, and aerobic exercises. Adherence was monitored using patients’ self-reported data.

Statistical analysis 

In the study, we used the SPSS version 15.0 (SPSS Inc., Chicago, IL) for data analysis. The statistical results are presented as the mean  ±  standard deviation (SD)/standard error, percentages, or median (minimum-maximum). Single-factor analysis of variance (ANOVA) was used, and a *P*-value < 0.05 was considered statistically significant.

## Results

Demographic characteristics of the study group are summarized in Table 1. Data are presented as mean ± SD.

**Table 1:** Demographic characteristics of the study group. SD, standard deviation; BMI, body mass index

Parameter	Mean± SD
Age (years)	65 ± 4.52
Female	66 ± 4.44
Male	65 ± 4.64
Height (cm)	1.70 ± 0.10
Weight (kg)	91 ± 14
BMI (kg/m^2^)	32.1 ± 2.82

Overall improvements were observed in waist circumference, BMI, and blood glucose among patients who participated in the physical activity program, as shown in Table 2.

**Table 2:** Baseline characteristics of the sample at study enrollment and after the physical activity program. SD, standard deviation

Baseline characteristics of the sample group	At study enrollment, mean ± SD	After the physical activity program, mean ± SD
Abdominal waist (cm)	102 ± 6.93	96 ± 5.53
Weight (kg)	91 ± 14	85 ± 14
Glycemia (mg/dl)	112 ± 10.21	109 ± 8.32

The mean ABI in the study sample was 0.98 before the physical activity program and 1.07 after the moderate physical activity program. The mean ABI for males was 0.95 at baseline and 1.03 at the end of the six-month physical activity program, while the mean ABI for females was 1.01 at baseline and 1.10 at the end of the study, as shown in Table 3.

**Table 3:** ABI of the sample at study enrollment and after the physical activity program. ABI, ankle-brachial index

ABI of the sample group	At study enrolment	After the physical activity program	*P* (*P* <0.05, significant, ANOVA)
Male ABI	0.95	1.03	<0.001
Female ABI	1.01	1.1	<0.001
Mean ABI	0.98	1.07	<0.001

Among participants in the study, patients with normal or low ABI at baseline showed a significant change in ABI after six months. In the study group, 11 men and 6 women had an ABI < 0.9 at the beginning of the study. After the six-month moderate physical activity program, 9 men and 6 women who had an ABI < 0.9 at the beginning of the study had an ABI > 0.9 at the end of the program. In participants with low ABI at baseline, we observed improvements in ABI values after they completed the prescribed moderate physical activity program, indicating an intervention effect. Fifteen of the 17 patients with abnormal ABI at the beginning of the study had a normal ABI at the end.

## Discussion

In June 2022, the *Annals of Internal Medicine* described the ABI as a noninvasive test that can be performed in a medical office and is more closely associated with leg function in individuals with PAD than intermittent claudication or other leg symptoms.

Our pilot study had some limitations. The physical activity program was assessed solely through patients’ self-reported data and was not a supervised exercise program, unlike those described by Gardner and Poehlman in 1995 and Watson et al. in 2005 [11,12], which have been shown to improve walking time and distance.

Exercise is beneficial, even among asymptomatic PAD patients, has been demonstrated by McDermott et al. in a randomized trial in patients with PAD with and without intermittent claudication [13]. It improves overall well-being and plays a preventive and rehabilitative role in patients with cardiovascular disease, as shown by Miller et al. [14]. Outcomes of supervised exercise programs are similar and longer lasting than surgical interventions [15]. Our pilot data address a relevant public health issue and use a practical primary care approach. The second goal of our study was to demonstrate the importance of implementing a moderate physical activity program in primary care for patients over 50 years, supported by preliminary data showing overall improvements in ABI in the sample group.

We aimed to promote a moderate physical activity program delivered by GPs to the general population over 50 years of age and to assess its effect on ABI measurements. ABI is a screening method used to identify PAD as well as overall cardiovascular risk. As shown in the results above, the ABI increased overall in the patient sample at the end of the six-month training program.

With a simple ABI assessment, we could improve patients’ awareness of the benefits of a moderate physical activity program, as they could see for themselves the changes in their ABI values.

The Cardiovascular Health Study, conducted by Siscovick et al., found that PAD diagnosed by ABI < 0.9 was more prevalent among older adults [16]. Although this study cannot assess temporality, its findings are consistent with ours.

In cross-sectional and longitudinal studies of older men, all physical activity and lower levels of sedentary behavior, regardless of duration, were inversely associated with ABI [17]. It is important to raise awareness of GP-led physical activity programs in the general population to prevent PAD. 

Our patients could benefit from a physical activity program delivered in primary care, potentially improving their ABI before PAD symptoms appear.

Adopting a physically active lifestyle is associated with a lower risk of developing peripheral arterial occlusive disease [18].

It is important to determine whether GPs should perform the ABI test on all patients rather than waiting for symptoms in those without risk factors. After assessing ABI in primary care, GPs could prescribe patients a routine moderate physical activity program, like the one used in our study, which may improve outcomes and help prevent some of the burden associated with established PAD.

In a study of 88 Romanians with metabolic syndrome, correlations of age, BMI, and lipid profile with endothelial dysfunction measured by brachial flow-mediated dilation and carotid intima-media thickness showed that these patients are at risk of developing coronary, cerebrovascular, and peripheral arterial disease [19]. Early implementation of ABI assessment and a routine moderate physical activity program could help improve their life expectancy.

The ABI test can be easily performed in primary care and may improve outcomes for patients with PAD.

Our study had limitations, including a relatively small sample size from a single clinic. No guidelines for measuring ABI were used. The moderate physical activity program was explained but not measured, so differences in program intensity among patients could have occurred. Adherence to the exercise program was self-reported, no control group was included, and follow-up was limited to six months. We did not perform long-term follow-up of the patients, which limits the reproducibility of our study.

## Conclusions

In all participants, ABI improvements were observed after the physical activity program. The physical activity program showed an additional effect on the ABI, as expected. Our study is a pilot intervention without a control group; while providing preliminary data, it highlights a relevant public health issue using a practical primary care approach. Launching a moderate physical activity program at the GP office would improve the ABI of asymptomatic patients with PAD. Promoting education and training of patients, particularly those over 50, at the GP office can raise awareness about the benefits of regular physical activity and the importance of monitoring ABI. This approach could prevent or delay PAD in the general population.
